# WORKFORCE DEVELOPMENT: AGING NETWORK CARE MANAGERS AS BEHAVIORAL HEALTH PROVIDERS FOR OLDER ADULTS IN COMMUNITIES

**DOI:** 10.1093/geroni/igac059.1846

**Published:** 2022-12-20

**Authors:** Mbita Mbao, Bronwyn Keefe

**Affiliations:** Salem State University, Salem, Massachusetts, United States; Boston University, Boston, Massachusetts, United States

## Abstract

**Background and Objectives:**

The population of older adults (65 and over) continues to increase with projections of one in five by 2030. Furthermore, one in four older adults have a behavioral health problem, and over 63% are not receiving behavioral health (BH) services. Many older adults living in the community depend on aging networks for home- and community-based services. However, most care managers' current education and training do not cover the skills and competencies to provide adequate care for older adults with BH needs. This study aimed to evaluate the effectiveness of a training intervention on the perceived self-efficacy of care managers working with older adults with BH issues in the aging network.

**Research Design and Methods:**

The study used a quasi-experimental design with a pre-and post-tests approach. The study used convenience sampling (n=90).

**Results:**

We found a significant difference in the mean self-efficacy scores related to working with clients with mental health problems between pre-test (M=62.31, SD=10.11) and post-test (M=65.88, SD=7.40). In addition, results indicated a significant difference in the mean self-efficacy score between the pre-test (M=59.81, SD=10.68) and post-test (M=65.60, SD=9.85) related to working with clients with substance use problems. Discussion and Implications: The study found that self-efficacy was higher at post-test than pre-test. In addition, there was no difference in the self-efficacy scores of those participants who had previously completed a mental health course or certification and those who had not.

